# Diet-induced shifts in the gut microbiota influence anastomotic healing in a murine model of colonic surgery

**DOI:** 10.1080/19490976.2023.2283147

**Published:** 2023-11-22

**Authors:** Sonja Boatman, Thomas Kaiser, Harika Nalluri-Butz, Mohammad Haneef Khan, Matthew Dietz, Julia Kohn, Abigail J Johnson, Wolfgang B Gaertner, Christopher Staley, Cyrus Jahansouz

**Affiliations:** a Department of Surgery, University of Minnesota, Minneapolis, MN, USA; b BioTechnology Institute, University of Minnesota, St. Paul, MN, USA; c School of Public Health, University of Minnesota, Minneapolis, MN, USA; d Division of Colon and Rectal Surgery, University of Minnesota, Minneapolis, MN, USA

**Keywords:** Gut microbiota, Colon surgery, anastomotic leak, mouse model, diet, fecal microbiota transplantation, leak index

## Abstract

Host diet and gut microbiota interact to contribute to perioperative complications, including anastomotic leak (AL). Using a murine surgical model of colonic anastomosis, we investigated how diet and fecal microbial transplantation (FMT) impacted the intestinal microbiota and if a predictive signature for AL could be determined. We hypothesized that a Western diet (WD) would impact gut microbial composition and that the resulting dysbiosis would correlate with increased rates of AL, while FMT from healthy, lean diet (LD) donors would reduce the risk of AL. Furthermore, we predicted that surgical outcomes would allow for the development of a microbial preclinical translational tool to identify AL. Here, we show that AL is associated with a dysbiotic microbial community characterized by increased levels of *Bacteroides* and *Akkermansia*. We identified several key taxa that were associated with leak formation, and developed an index based on the ratio of bacteria associated with the absence and presence of leak. We also highlight a modifiable connection between diet, microbiota, and anastomotic healing, potentially paving the way for perioperative modulation by microbiota-targeted therapeutics to reduce AL.

## Introduction

Inflammatory bowel disease, cancer, and diverticulitis are common diseases of the colon with an estimated 5–15% lifetime risk of development.^1–3^ Surgical resection and anastomosis (healthy bowel connection) is often necessary for successful treatment of colorectal disease; accordingly, more than 600,000 colectomies are performed annually in the United States.^1–3^ However, serious complications occur in up to 20% of surgeries from anastomotic leak (AL) resulting in significant patient morbidity and mortality plus an increased length of hospital stay and economic burden.^4–10^ While clinical outcome studies investigating comorbidities and surgical factors have revealed important elements influencing patient outcomes,^4,11–13^ they have reached a point of diminishing return, emphasizing the urgent need to identify patients at risk for AL.

Surgical technique is critical in creating a healthy, well-perfused and tension-free anastomosis. However, AL and surgical site infections (SSI) still occur with optimal surgical technique, and surgeons cannot accurately predict which patients will develop AL.^14^ Many studies have been performed to identify risk factors for AL and SSI given the severity of its consequences.^11–13^ Even when risk factors are deemed modifiable, improving these risk factors in the 4 to 6 weeks between clinic evaluation and surgery is a difficult, if not impossible, task.

The gut microbiota comprises an ecosystem that is vital to intestinal health through its numerous functions, including energy harvest, metabolite production, and induction of pro- and anti-inflammatory immune responses.^15,16^ It has been shown that the interaction of host factors, such as diet, and the gut microbiota, directly impacts gut recovery, wound healing, and AL.^17–20^ Microbial compositional shifts dominated by a collagenolytic strain of *Enterococcus faecalis*, which is associated with impaired tissue healing, have been demonstrated in mice fed an obesogenic diet, leading to increased rates of AL.^21–23^ Notably, there is a paucity of studies investigating the impact of a combination high-fat and high-sugar diet, which is more representative of the post-agricultural “Western diet” (WD),^24^ on the relationship between gut microbial composition and AL. WD is known to cause deleterious shifts in the microbiome toward a pro-inflammatory profile mediated by diminished short-chain fatty acid (SCFA) production and decreased primary bile acid deconjugation.^16,25^ This is relevant given the rising rate of colorectal diseases, including cancer and diverticulitis, not only in the United States, but also globally with the spread of WD to developing nations.^26,27^ Thus, investigating mechanisms by which diet alters the gut microbiota represents a critical need that may allow the creation of tools that identify patients pre-operatively who are at higher risk for these devastating surgical complications. Interventions that increase protective commensal bacteria that increase microbiome diversity and reduce pathogenic inflammation may mitigate intestinal-microbiota driven complications.^28^ Furthermore, the microbiome is highly and rapidly modifiable, making it an attractive therapeutic target, in contrast to most patient comorbidities.^29–31^

**Figure 1.**
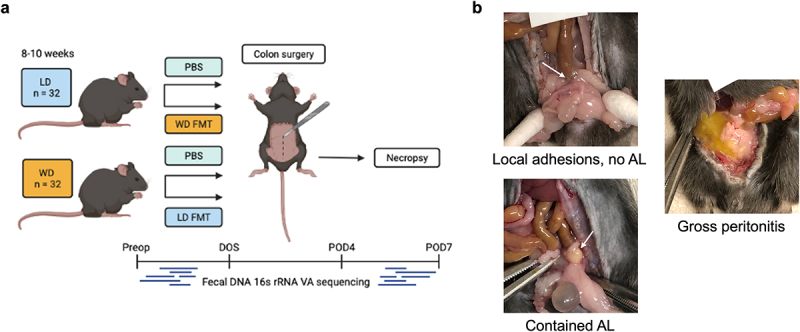
(a) Schematic of experimental protocol. (b) Anastomotic scoring examples demonstrating healed anastomosis with local adhesions (top left), contained AL/perianastomotic abscess (bottom left), and anastomotic dehiscence with gross peritonitis (right). Arrows point to anastomosis.

The goal of this study was to investigate if diet was a mechanism by which microbiota may be modified to affect AL in a murine model of colonic anastomosis. Thus, mice were either introduced to lean diet (LD) or Western Diet (WD). In order to determine the influence of the microbiota on anastomotic leak occurrence, reciprocal fecal microbiota transplantation (FMT) was performed whereby a cohort of WD-fed mice received an LD FMT (WD-ldFMT) and a cohort of LD-fed mice received a WD FMT at the time of surgery (LD-wdFMT) (Figure 1). We predicted that diet would impact gut microbial composition and resultant changes would correlate with AL risk, and LD-wdFMT mice would have an increased risk of AL while WD-ldFMT would have a reduced risk of AL. Furthermore, we predicted that the surgical outcomes from the four variable murine groups would allow for the development of a microbial preclinical translational tool that may predict AL.

## Results

### Effect of diet on clinical endpoints

#### Survival

Of 64 mice, 59 (92%) survived until necropsy (Table 1). All surviving mice demonstrated full clinical recovery with tolerance of post-operative diet, return of bowel function, and lack of signs of infection. Among mice that did not survive until postoperative day (POD) 7, 3 mice died from anesthesia and 2 mice were euthanized early for various reasons (anastomosis was intact for both). No significant differences in survival were found between dietary groups (*P* = 0.38, Fisher’s exact test) (Table 1).

**Table 1.** Survival contingency table: no significant differences in survival to necropsy on POD7 between groups.

	Survived (n)	Died (n)	Fisher’s exact test *P*-value
LD PBS	15	1	0.38
WD PBS	15	1	
WD-ldFMT	16	0	
LD-wdFMT	13	3	

#### Anastomotic leak

All leaks were contained with peri-anastomotic abscess without gross contamination. As such, anastomoses were categorized as either having the presence or absence of leak. Overall leak rates in the control groups were consistent with that observed by others.^32,33^ LD and WD mice had a similar leak rate: 6/15 (40%) of LD and 5/15 (33%) of WD mice had AL (*P* = 0.71, Chi-square test) (Table 2). LD mice that received WD FMT had no significant change in leak rate: 4/16 (25%) had AL (*P* =.46, vs. PBS controls, Fisher’s exact test) (Table 2). Conversely, WD mice that received LD FMT had a significant reduction in AL: 0/13 mice had AL (*P* =.044, vs. PBS controls, Fisher’s exact test) (Table 2).

**Table 2.** AL contingency table: no significant difference in AL observed between control dietary groups or between LD mice that received PBS vs. FMT. Significant reduction in AL observed in WD mice that received FMT vs. PBS.

	No AL (n)	+AL (n)	Chi-square or Fisher’s exact test *P*-value*
LD PBS	9	6	0.71
WD PBS	10	5	
LD PBS	9	6	0.46
LD-wdFMT	12	4	
WD PBS	10	5	0.04
WD-ldFMT	13	0	

*Fisher’s exact test used if contingency table contains cells with value < 5.

#### Body weight

**Figure 2.**
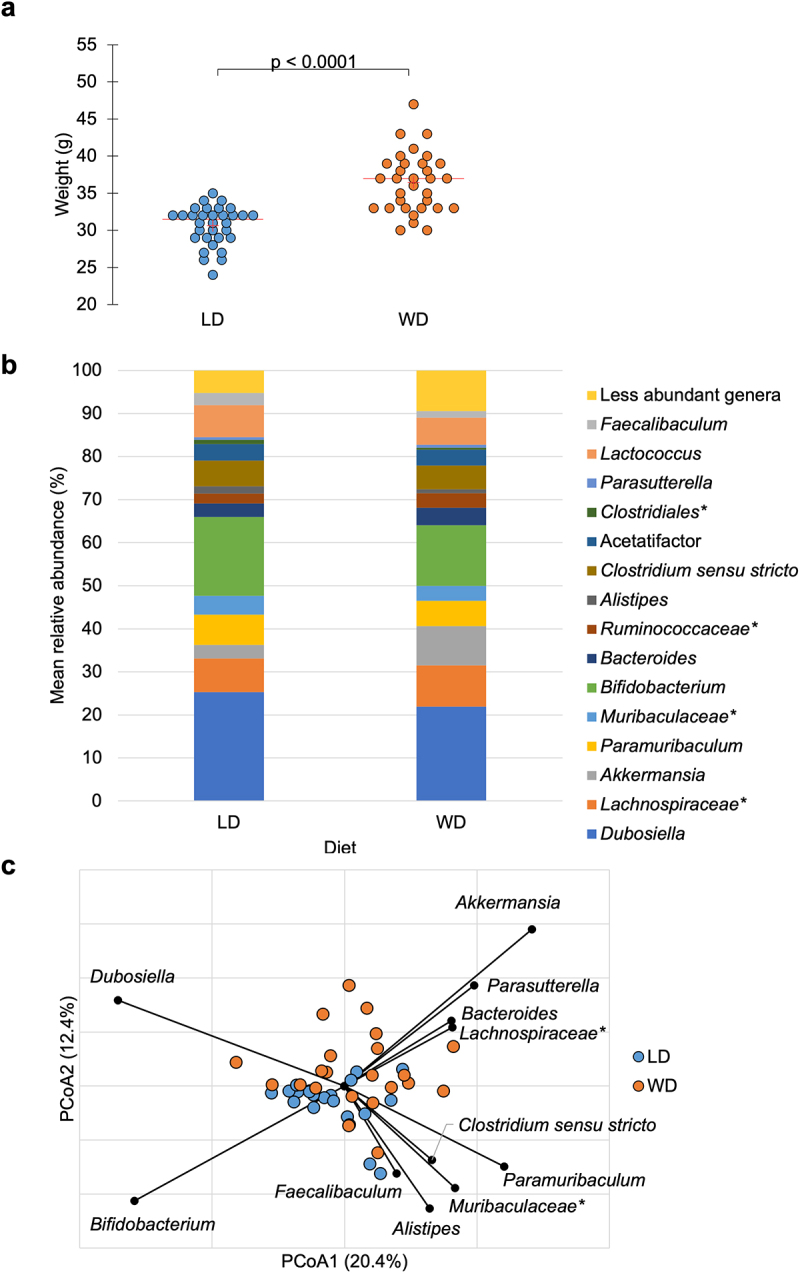
a) Mean weight on DOS varied by dietary group: LD: 30.6 ± 2.6 g, WD: 36.4 ± 4.0 g (ANOVA F = 47.2, *P* <.0001). b) abundant taxa for each dietary groups after 10-week feeding period prior to DietGel initiation (baseline). c) Bray-Curtis dissimilarity indices demonstrated significant clustering by diet at baseline (ANOSIM *R* = 0.15, *P* <.001). Genera significantly correlated to dietary axis position by Spearman correlation are plotted on the PCoA chart with vector length indicating magnitude of correlation. *Not further classified to genus.

After the 10-week preoperative feeding period, mean body weight on the day of surgery (DOS) was significantly different between dietary groups: 30.6 ± 2.6 g vs. 36.4 ± 4.0 g for LD and WD mice, respectively (F = 47.2, *P* <.0001, analysis of variance [ANOVA]) (Figure 2a).

### Dietary impact on intestinal microbiota

Alpha diversity was significantly higher in the LD compared to WD mice at baseline, with mean Chao1 index of 172.6 ± 4.8 for LD and 150.6 ± 4.7 for WD (F = 10.7, *P* = .0022, ANOVA) (Table 3). Alpha diversity was also significantly higher on POD4 in LD mice, with mean Shannon index of 3.2 ± 0.05 vs. 3.0 ± 0.05 (F = 6.5, *P* = .014, ANOVA) and mean Chao1 index of 202.2 ± 11.3 vs. 166.9 ± 12.3 (F = 4.5, *P* =.039, ANOVA) for LD and WD, respectively (Table 3).

**Table 3.** Alpha diversity. ANOVA performed for pairwise comparisons demonstrated that LD had significantly higher alpha diversity at baseline (by Chao1 index) and on POD4 (by Shannon and Chao1 index). On POD4, WD mice with AL had significantly lower alpha diversity compared to mice without AL in both PBS and FMT groups.

		Mean Shannon index ± SE (a, b)*					Mean Chao1 index ± SE (a, b)*				
		LD		WD		ANOVA P-value	LD		WD		ANOVA P-value
Baseline		2.7 ± 0.06 (a)		2.8 ± 0.06 (a)		0.19	**172.6 ± 4.8 (b)**		**150.6 ± 4.7 (a)**		**0.0022**
DOS		2.8 ± 0.05 (a)		2.7 ± 0.05 (a)		0.27	180.0 ± 6.1 (a)		170.6 ± 6.5 (a)		0.32
POD4		**3.2 ± 0.05 (b)**		**3.0 ± 0.05 (a)**		**0.014**	**202.2 ± 11.3 (b)**		**166.9 ± 12.3 (a)**		**0.039**
POD7		3.0 ± 0.04 (a)		3.1 ± 0.05 (a)		0.36	185.6 ± 18.0 (a)		199.0 ± 18.9 (a)		0.61
		AL – No		AL – Yes		P-value	AL – No		AL – Yes		P-value
LD	Baseline	2.7 ± 0.08 (a)		2.6 ± 0.10 (a)		0.69	171.4 ± 6.5 (a)		174.6 ± 8.3 (a)		0.77
	DOS	2.7 ± 0.06 (a)		2.8 ± 0.08 (a)		0.47	177.6 ± 5.5 (a)		183.8 ± 8.0 (a)		0.53
	POD4	3.2 ± 0.06 (a)		3.0 ± 0.08 (a)		0.067	213.3 ± 18.3 (a)		179.0 ± 26.5 (a)		0.30
	POD7	3.0 ± 0.05 (a)		3.1 ± 0.08 (a)		0.25	190.5 ± 12.4 (a)		175.3 ± 17.9 (a)		0.49
WD	Baseline	2.7 ± 0.07 (a)		2.8 ± 0.13 (a)		0.52	150.0 ± 5.2 (a)		152.3 ± 9.6 (a)		0.84
	DOS	2.7 ± 0.05 (a)		2.8 ± 0.11 (a)		0.33	173.5 ± 8.9 (a)		157.6 ± 18.7 (a)		0.45
	POD4	**3.0 ± 0.05 (b)**		**2.7 ± 0.10 (a)**		**0.0023**	167.7 ± 3.3 (a)		163.8 ± 6.7 (a)		0.61
	POD7	3.1 ± 0.05 (a)		3.2 ± 0.10 (a)		0.27	208.2 ± 27.9 (a)		156.6 ± 59.9 (a)		0.44
POD4		PBS		FMT		P-value	PBS		FMT		P-value
LD		AL – No	AL – Yes	AL – No	AL – Yes		AL – No	AL – Yes	AL – No	AL – Yes	
		3.2 ± 0.09 (a)	3.0 ± 0.11 (a)	3.2 ± 0.08 (a)	3.1 ± 0.13 (a)	0.25	229.2 ± 28.7 (a)	180.0 ± 35.1 (a)	201.4 ± 24.8 (a)	177.5 ± 43.0 (a)	0.66
WD		**3.1 ± 0.08 (b)**	**2.7 ± 0.10 (a)**	**3.0 ± 0.07 (b)**	-	**0.0085**	174.7 ± 4.8 (a)	163.8 ± 6.4 (a)	162.4 ± 4.1 (a)	-	0.15

*Pairwise comparisons are denoted by lower case letters, (a) <; (b). Bolded values in the table are statistically significant.

Beta diversity was compared among the dietary groups, and significant clustering by diet was observed at baseline (analysis of similarity [ANOSIM] *R* = 0.15, *P* < 0.001) (Figure 2b, c). This pattern remained on DOS, POD4 and POD7, with separation of LD and WD communities at all timepoints tested (ANOSIM *R* ≥ 0.094, *P* < .001). Spearman correlation tests were performed to determine genera associated with principal coordinate analysis (PCoA) axis positions between diets at baseline (Figure 2c). *Bacteroides*, *Akkermansia*, *Parasutterella, Dubosiella*, and *Lachnospiraceae* spp. were significantly associated with WD axis position (*P* ≤0.04), while *Paramuribaculum*, *Clostridium sensu stricto, Alistipes, Bifidobacterium, Faecalibaculum*, and members of family *Muribaculacaeae* were significantly associated with LD axis position (*P* ≤0.04). Kruskal–Wallis pairwise comparisons of the predominant genera among dietary groups at baseline were performed. Of the genera significantly associated to diet by Spearman correlation, *Akkermansia* had higher relative abundance in the WD vs. LD group (9.1 ± 5.9% vs. 3.1 ± 3.6%, *P* <.001) and *Alistipes* was more abundant in the LD vs. WD group (1.7 ± 0.65% vs. 0.88 ± 0.86%, *P* <.001). Linear discriminant analysis (LDA) effect size (LEfSe) was also performed to further investigate taxa associated with diet at baseline, demonstrating that *Akkermansia* had greater relative abundance in WD (LDA = 4.5, *P* =.0007).

### Microbiota changes associated with AL in PBS control mice

Among LD mice, alpha diversity was not significantly different between mice with and without AL at any timepoint (*P* ≥0.067 for all). However, alpha diversity was significantly greater in WD-fed mice without AL compared to those with AL on POD4 (mean Shannon index 3.0 ± 0.05 vs. 2.7 ± 0.10, F = 11.7, *P* =.0023) (Table 3).

**Figure 3.**
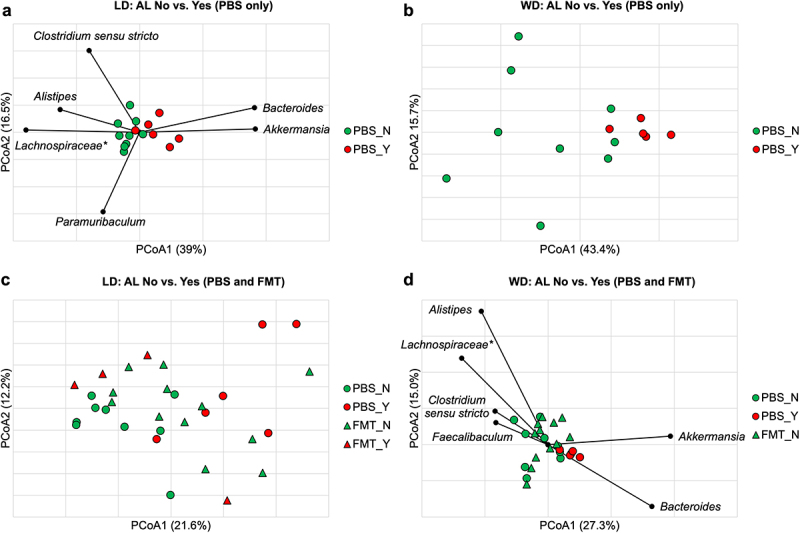
(a) Significant clustering was observed on POD4 in the LD mice based on presence or absence of AL (ANOSIM *R* = 0.49, *P* <.001) with genera significantly correlated to axis position by Spearman correlation shown. (b) clustering between AL and no AL approached significance in the WD group (ANOSIM *R* = 0.27, *P* =.05). (c) clustering based on AL was lost with in LD mice that received FMT (FMT_N vs. FMT_Y, ANOSIM *R* = 0.085, *P* = 0.26). (d) significant clustering between AL and no AL was demonstrated among WD mice (ANOSIM *R* = 0.15, *P* =.047). FMT treatment made mice without AL further from mice with AL than PBS (FMT_N vs. PBS_Y, ANOSIM *R* = 0.28, *P* = 0.017; PBS_N vs. PBS_Y, ANOSIM *R* = 0.27, *P* =.043, Bonferroni pair-wise error rate 0.017). Genera significantly correlated to axis position by Spearman correlation are shown. *Not further classified to genus.

Beta diversity was compared between mice with and without AL within each dietary control group using Bray-Curtis dissimilarity indices. There was no clustering at baseline or on DOS related to AL (ANOSIM *R* ≤ 0.11, *P* ≥0.14). Significant clustering was seen on POD4 (ANOSIM *R* = 0.49, *P* <.001) (Figure 3a) and POD7 (ANOSIM *R* = 0.25, *P* =.03) in the LD mice based on the presence or absence of AL. This approached significance in the WD group on POD4 (ANOSIM *R* = 0.27, *P* =.05) (Figure 3b), but not on POD7 (ANOSIM *R* = 0.011, *P* =.44). Spearman correlation tests of LD mice on POD4 showed *Akkermansia* and *Bacteroides* were significantly associated with the axis position of mice with AL (*P* ≤0.001), while *Alistipes, Clostridium sensu stricto, Paramuribaculum*, and *Lachnospiraceae* spp. were associated with no AL (*P* ≤0.02) (Figure 3a). Kruskal–Wallis pairwise comparisons demonstrated that *Alistipes, Clostridium sensu stricto*, and *Lachnospiraceae* spp., were present in significantly greater relative abundances in LD control mice without vs. with AL on POD4 (8.2 ± 2.4% vs. 3.9 ± 1.9%, 4.3 ± 2.0% vs. 2.0 ± 0.93%, and 17.0 ± 6.1% vs. 9.9 ± 6.1%, respectively, *P* < 0.05), while there were greater abundances of *Akkermansia* and *Bacteroides* in mice with vs. without AL (20.6 ± 10.4% vs. 9.5 ± 2.9% and 10.1 ± 3.8% vs. 2.9 ± 2.0%, respectively, *P* ≤0.01). LEfSe showed that *Alistipes* had a greater relative abundance in mice without AL (LDA = 4.1, *P* =.007) and *Akkermansia* and *Bacteroides* had greater relative abundances in mice with AL (LDA >4.5, *P* <.005). In LD controls on POD7, similar genera were associated with AL by Spearman correlation as on POD4, however no genera were found to be significantly different between AL and no AL groups on Kruskal–Wallis analysis. As such, because the difference in community composition based on AL was strongest on POD4, further analyses investigating an AL signature were performed on fecal microbiota obtained at this timepoint.

### Relationship of dietary macronutrients to genera associated with AL

**Figure 4.**
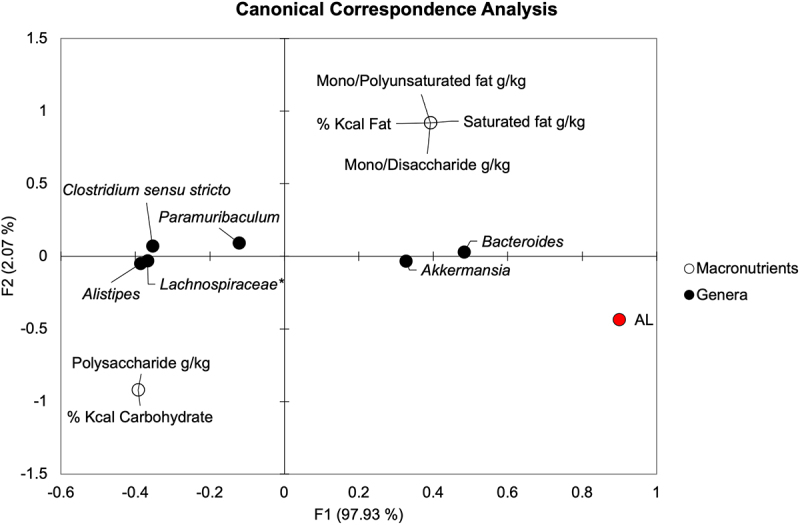
Canonical correspondence analysis demonstrates directional relationships between macronutrients, AL, and genera on POD4. *Not further classified to genus.

We sought to determine if specific macronutrients were correlated with genera, particularly those associated with AL. Canonical correspondence analysis (CCA) with Spearman correlation was performed comparing dietary carbohydrate and fat percentages, amounts of simple and complex sugars, and saturated and unsaturated fat quantities to AL and genera significantly associated with AL (Figure 4). On POD4, Spearman correlation showed that *Alistipes*, *Clostridium sensu stricto, Paramuribaculum*, and *Lachnospiraceae* spp. were negatively correlated with AL (*R* = −0.60, −0.60, −0.47, and −0.60, respectively, *P* ≤.011). Meanwhile, *Akkermansia* and *Bacteroides* positively correlated with AL (Spearman *R* = 0.60 and 0.65, respectively, *P* <.001). Out of these genera, only *Bacteroides* showed a significant directional relationship with dietary macronutrients. *Bacteroides* correlated positively with total fat percentage, saturated fat, mono- and polyunsaturated fat, and simple sugar content (Spearman *R* = 0.45, *P* =.01 for all) and negatively with total carbohydrate percentage and polysaccharide content (Spearman *R* = −0.45, *P* =.01 for both). These results suggest that there may be an indirect relationship of macronutrients on AL that is mediated by specific bacterial genera, such as *Bacteroides* in the setting of a high fat, high-simple sugar WD.

### Impact of FMT on AL

FMT disrupted the microbial relationship with AL in the LD group. Unlike the PBS controls where AL status significantly differentiated community composition, LD mice receiving FMT (LD-wdFMT) had no significant differences in community composition based on AL status at any timepoint tested (ANOSIM *R* ≤ 0.25, *P* ≥.20). Communities from the LD-wdFMT mice without AL clustered separately from PBS controls without AL (ANOSIM *R* =.20, *P* =.007) (Figure 3c) but could not be differentiated from control or LD-wdFMT mice with AL (ANOSIM *R* = 0.15 and 0.085, *P* =.12 and 0.26, respectively) (Figure 3c), suggesting receipt of WD fecal slurry shifted the community in favor of an AL-promoting assemblage. Conversely, WD mice that received FMT (WD-ldFMT) had no leaks. While differences in community composition between AL and no AL approached statistical significance in WD control mice (ANOSIM *R* = 0.27, *P* =.05) (Figure 3b), FMT was found to increase the dissimilarity between WD-ldFMT mice (no AL) and WD control mice with leak (ANOSIM *R* = 0.28, *P* = 0.03) (Supplemental Figure S1). *Akkermansia* and *Bacteroides* remained significantly associated with the axis position of WD mice with AL when FMT groups were included (Spearman *P* <.0001) and *Alistipes, Clostridium sensu stricto, Lachnospiraceae* spp., and *Faecalibaculum* were directionally associated with no AL (Spearman *P* <.04) (Figure 3d).

To verify engraftment among the FMT groups, we used the Bayesian algorithm SourceTracker2 (Supplemental Figure S2). Among the LD-wdFMT group, the WD fecal slurry was found to engraft at 33.0 ± 10.9%, 11.8 ± 6.2%, and 15.5 ± 8.0% of the community, with respect to DOS, POD4, and POD7. Among the WD-ldFMT group, the LD fecal slurry engrafted at 10.6 ± 9.3%, 5.1 ± 9.2%, and 9.8 ± 5.2%, with respect to the time of sampling. Pooling both FMT groups, engraftment was significantly greater on DOS than POD4 or POD7 (ANOVA F = 20.423, Tukey’s *post-hoc P* <.0001), although no differences in engraftment were observed between POD4 and POD7 (*post-hoc P* = 0.076), and no significant temporal changes in engraftment were observed in the WD-ldFMT group. The WD fecal slurry also engrafted to a greater extent in LD-wdFMT mice than the LD fecal slurry in WD-ldFMT mice (*P* < 0.0001). No differences in engraftment between the LD-wdFMT and WD-ldFMT groups were observed on POD4 or POD7 (*post-hoc P* =.232 and 0.159, respectively).

### Identifying a microbial signature associated with AL

There were no significant differences in community composition observed between mice that were found to have AL, regardless of diet group (ANOSIM *R* = 0.29–0.84, *P* ≥.008 at Bonferroni corrected α = 0.002). Due to the similarity in community composition among mice that had AL, these mice were grouped for subsequent analysis. The five main comparison groups thus consisted of all mice with AL and mice without AL from the four treatment groups; subsequent reference to LD, LD-wdFMT, WD, and WD-ldFMT includes only mice without AL.

**Figure 5.**
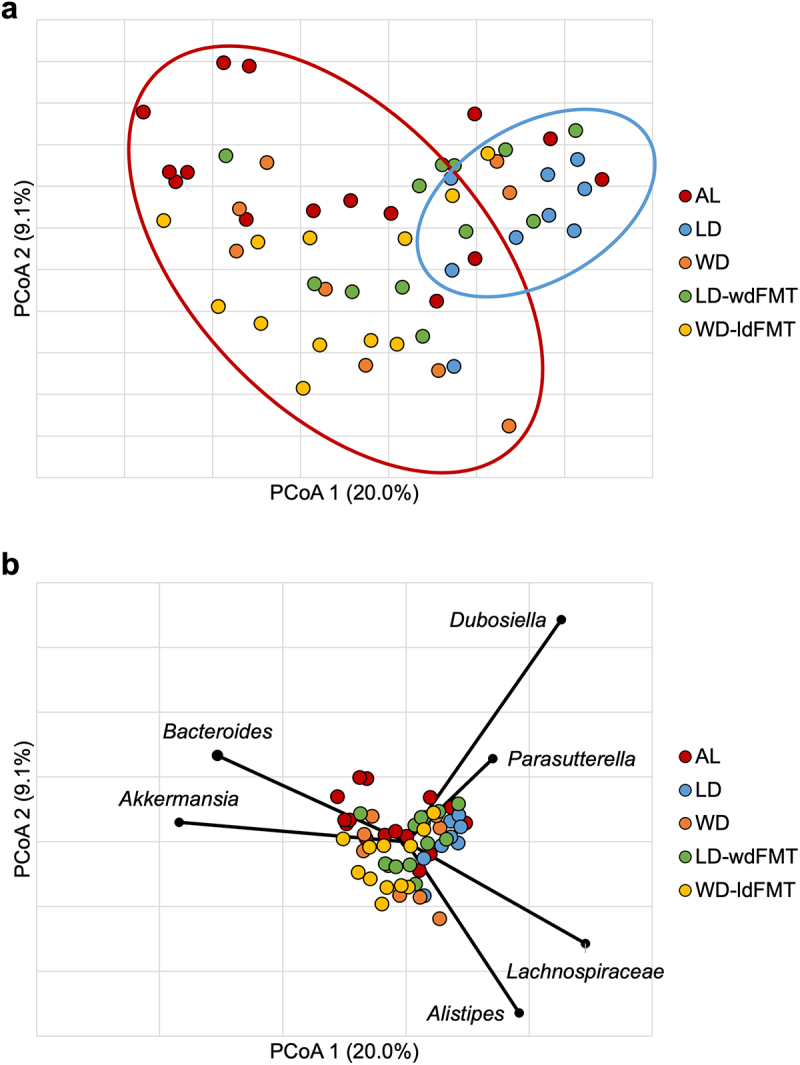
(a) Principal coordinate analysis (r^2^ = 0.67) of Bray-Curtis dissimilarities among all samples on POD4. Groups are differentiated by color. Ellipses indicate approximate separation of samples by ANOSIM. (b) genera significantly correlated to axis position by Spearman correlation. *Not further classified to genus.

Alpha diversity was not significantly different between groups with a mean Shannon index 3.1 ± 2.7 (F = 2.401 *P* ≥.059). Further community analysis showed significant differences between the AL and LD groups (ANOSIM *R* = 0.33, *P* =.002, Bonferroni corrected α = 0.005). A significant difference was noted between groups fed WD (WD and WD-ldFMT) and groups fed LD (LD and LD-wdFMT) (R 0.28–0.48, *P* ≤.003, Bonferroni corrected α = 0.005) (Figure 5a). However, no significant differences were noted between the AL group and the WD, WD-ldFMT, or LD-wdFMT groups (*R* = −0.01–0.17, *P* ≥.009, Bonferroni corrected α = 0.005). Given the majority of groups did not have significant community differences from the AL group, specific taxa that may be associated with AL were investigated.

### Taxa associated with AL

Spearman correlation tests were performed to determine genera associated with AL (Figure 5b). *Bacteroides*, *Akkermansia*, *Dubosiella*, *Lachnospiraceae* spp., *Alistipes*, and *Parasutterella* were significantly associated with axis position (*P* ≤.007), with *Bacteroides* and *Akkermansia* being most closely associated with AL samples. Kruskal–Wallis pairwise comparisons of the predominant genera among various groups and the AL group were performed (Table 4). *Paramuribaculum*, *Clostridium sensu stricto*, and *Faecalibaculum* were also identified as potentially associated with AL. Finally, LEfSe analysis indicated that *Paramuribaculum* and *Clostridium sensu stricto* had greater relative abundances in WD and WD-ldFMT. Conversely, communities in mice from the LD and LD-wdFMT groups harbored greater relative abundances of *Alistipes* and the AL group had greater abundances of *Bacteroides* and *Akkermansia* (Supplemental Figure S3).

**Table 4.** Median relative abundances of predominant genera with interquartile range in brackets. Bolded taxa indicate a significant difference way found in a Kruskal–Wallis pairwise comparison. Superscripts indicate which specific Kruskal–Wallis pairwise comparison was significant.

Taxa	AL	LD	LD-wdFMT	WD	WD-ldFMT
*Acetatifactor*	1.7 [1.32–2.96]	1.67 [1.37–1.93]	1.71 [1.58–2.12]	2.13 [1.91–3.29]	2.62 [1.8–3.33]
*Akkermansia*	17.72 [13.05–28.9]^b^	9.93 [7.44–10.41]^b^	13.21 [11.28–16.57]^b^	17.67 [8.14–19.71]	16.39 [11.01–21.5]
*Alistipes*	4.39 [2.69–6]^abc^	7.31 [6.35–10.35]^b^	7.01 [6.33–8.61]^b^	6.05 [3.58–7.4]	6.41 [5.03–7.72]^c^
*Bacteroides*	9.9 [6.84–14.25]^ab^	2.16 [1.85–4.25]^b^	6.28 [5.58–8.43]^b^	6.55 [4.37–9.97]	7.45 [6.32–9.02]
*Bifidobacterium*	0.52 [0.38–1.39]	1.06 [0.34–1.72]	1.79 [0.67–2.57]	0.48 [0.16–0.72]	0.35 [0.07–0.69]
Clostridiales*	3.42 [2.13–4.6]	4.23 [1.8–4.99]	3.58 [1.95–4.17]	2.33 [0.78–4.36]	4.41 [2.78–6.45]
*Clostridium sensu stricto*	1.93 [0.57–2.2]^abc^	4 [3.83–4.99]^b^	2.09 [0.05–2.8]^b^	3.72 [2.37–4.88]	4.1 [2.13–4.45]
*Dubosiella*	14.97 [11.94–18.62]^c^	22.24 [18.27–24.5]	16.54 [10.28–17.82]	12.32 [10.91–15.63]	10.14 [5.51–14.1]^c^
*Faecalibaculum*	0 [0–0]^c^	0 [0–0]	0 [0–0]	0 [0–0]	0.05 [0.02–0.57]^c^
Lachnospiraceae*	9.72 [4.37–13.61]^ab^	15.35 [14.05–17.94]^b^	15.65 [13.4–18.85]^b^	12.31 [10.14–16.2]	11.08 [8.4–12.62]
*Lactococcus*	0 [0–0]	0 [0–0]	0 [0–0]	0 [0–0]	0 [0–0]
Muribaculaceae*	9.14 [8.12–10.53]	8.92 [5.72–9.8]	8.27 [7.02–10.22]	8.14 [6.3–8.28]	8.83 [6.69–9.86]
*Paramuribaculum*	7.93 [6.58–8.49]^ac^	8.15 [7.26–9.94]	8.44 [5.95–9.27]	11.3 [9.97–12.48]	10.95 [9.58–12.02]^c^
*Parasutterella*	1.37 [0.98–1.47]	1.63 [1.41–1.75]	1.08 [0.93–1.59]	1.29 [1.02–1.43]	0.92 [0.76–1.5]
Ruminococcaceae*	4.71 [3.67–5.45]	4.06 [3.63–4.85]	4.92 [4.41–7.08]	4.58 [3.79–5.57]	5 [3.45–7.58]

^a^Comparisons between all samples without AL vs AL samples.

^b^Comparisons between LD and LD-wdFMT samples compared to the AL samples.

^c^Comparisons between WD-ldFMT samples and AL samples.

*Not further classified to genus.

### Identifying predictive taxa

From the previous analysis, 8 taxa had been identified as associated with AL: *Dubosiella*, *Lachnospiraceae* spp., *Akkermansia*, *Paramuribaculum*, *Bacteroides*, *Alistipes*, *Clostridium sensu stricto*, and *Parasutterella*. These 8 taxa were selected to develop predictive classification and regression trees (CART) with entropy (information gain) used as a quality measure. Samples were randomized and split equally into either a training dataset (*n* = 18–29) or prediction dataset (*n* = 18–28) with presence of AL as the dependent outcome. A CART containing all the groups selected *Lachnospiraceae* spp., *Parasutterella*, and *Bacteroides* as predictive genera, however, its overall accuracy was 57% with a sensitivity of 50% and specificity of 59%. Given that groups fed WD (WD and WD-ldFMT) had separate communities compared to groups fed LD (LD and LD-wdFMT), CARTs investigating these groups were created separately. A CART containing groups that were fed WD (WD and WD-ldFMT) and AL selected *Paramuribaculum*, *Dubosiella*, and *Clostridium sensu stricto* as predictive, with overall accuracy of 89%, sensitivity of 83% and specificity of 92%. Finally, a CART containing groups that were fed LD (LD and LD-wdFMT) and AL selected *Clostridium sensu stricto*, *Alistipes*, and *Dubosiella*, with overall correct prediction rate of 72%, sensitivity 89% and specificity of 56%.

#### AL index

Based on iterative CART analyses, greater relative abundances of *Paramuribaculum, Clostridium sensu stricto*, and *Alistipes* were highly discriminatory for absence of AL whereas *Dubosiella* and *Bacteroides* were predictive of the presence of AL. Given the potential predictive power of these genera, an AL index was created using the relative abundances of the above genera as well as *Akkermansia*, which was selected given its significant Spearman correlation with AL and association with AL on LEfSe. The AL index uses the following ratio based on selected genera’s relative abundance:

$$\frac{Paramuribaculum+Clostridium\;sensu\;stricto+Alistipes}{Dubosiella+Bacteroides+Akkermansia}$$

Given the previous evidence, it was predicted that a lower value on the index would indicate increased risk of a leak. When applying this AL index to the entire dataset with cutoff of 0.45 predicting the absence of leak (Table 5), the overall correction prediction rate was 73%, sensitivity 80%, and specificity 71%. When comparing the AL index between groups, LD and WD-ldFMT were found to have a significantly greater index when compared to AL (Dunn’s *post-hoc p* ≤.003, Bonferroni corrected α = 0.005) (Table 5).

**Table 5.** Median AL index scores among all groups with interquartile range in brackets.

Group	AL Index
LD	0.59 [0.56–0.71]^a^
LD-wdFMT	0.47 [0.4–0.63]
WD	0.53 [0.4–1.01]
WD-ldFMT	0.55 [0.45–0.72]^a^
AL	0.31 [0.2–0.43]^a^

^a^Significant difference found in Kruskal-Wallis pairwise comparison.

## Discussion

Previous studies have shown that leak rate is impacted by diet and the microbiome, with WD increasing the risk of AL and nutritional therapy decreasing risk.^21,34^ In this study, we aimed to investigate the effects of microbiota manipulations, specifically with diet and FMT, on AL. Our objective was to identify diet-influenced patterns of microbiota associated with AL, and if these genera could be altered via FMT to influence clinical outcomes. Our results indicated that several specific genera may be mechanistically linked, since the overall microbial community compositions of mice with AL displayed no significant differences on the basis of their dietary group. Conversely, we observed unique microbial communities in mice without leaks in each dietary group. Our analysis suggests that mice with AL have a dysbiotic microbial community, which is consistent with work that suggests dysbiosis increases leak risk.^35,36^ Here, we offer a novel, translational index linking six genera with both putative protective and pathological effects and capture potential inter-species interactions that we will explore mechanistically in future investigations. Moreover, we demonstrate that correction of this microbial dysbiosis by FMT may be protective of AL in our murine model.

Perioperative dietary modification is widely employed by surgeons, traditionally consisting of preoperative fasting and gradual postoperative diet advancement, though accelerated diet advancement is becoming more common in the era of enhanced recovery pathways.^37^ In a study by Hyoju et al.,^21^ microbial shifts and worse anastomotic healing were observed in mice fed a high-fat/low-fiber diet compared to low-fat/high-fiber standard chow. The authors further demonstrated that mice on a high-fat/low-fiber diet that were rehabilitated with a 2-day crossover period of standard chow prior to surgery had relative abundances of bacterial phyla resembling standard-chow control mice and significant improvement in anastomotic healing scores. In line with this previous study, we elected to directly and rapidly induce preoperative microbial effects with FMT on the DOS. FMT is a clinically feasible therapy that has been adopted as an effective treatment for recurrent *Clostridioides difficile* infection and is being explored in various settings including inflammatory bowel disease, metabolic syndrome, and diabetes.^29,38–40^

FMT with LD fecal slurry had a protective effect on anastomotic healing, with no leaks observed in the WD group receiving FMT. FMT-treated WD mice also had a concomitant shift in their microbial community away from the AL assemblage seen in control mice. This demonstrates a specific microbial driven effect of FMT on AL, potentially via reduction of dysbiosis and restoration of a healthy microbiome amidst the stress of surgery. There was a shift in the microbiome of FMT-treated LD mice without leak toward the AL assemblage, indicating that WD FMT induced dysbiosis; however, there was no difference in leak rate among LD mice regardless of FMT. Microbial perturbation may not have impacted clinical outcomes in LD mice due to the baseline metabolic fitness of the non-obese animal. Notably, control LD and WD mice had no difference in AL rate despite their distinct microbial community compositions and the dysbiosis of WD mice. It is possible that the physiologic stress of surgery and disruptions of the intestinal microbiome in the immediate perioperative period outweighed any clinical impact of baseline microbiome differences between LD and WD, and this warrants further investigation. Nonetheless, our observed reduction of AL in WD-fed mice after receipt of LD fecal slurry in this preclinical model lays the foundation for future larger-scale animal studies and the potential role of perioperative FMT in the clinical setting.

Despite differences in specific taxa involved in leak prediction, the ratio of taxa associated with the absence to the presence of AL was found to be informative in our data. This suggests that an index could be developed for humans to detect leaks in their early, asymptomatic stages and potentially treat them before they become more severe and clinically detectable.^41^ This is consistent with other studies that have evaluated the predictive potential of the gut microbiota in detecting other post-surgical complications as well as the presence of diseases. Specifically, gut microbial composition has been correlated with higher post-operative complications after pancreatic surgery and the presence of metachronous adenoma formation in colorectal cancer patients following surgery.^42,43^ Patterns of microbiota disruption, specifically loss of diversity, have also been shown to predict poor survival following allogeneic hematopoietic-cell transplantation.^44^ Using gut microbial markers as a tool, Ren et al.^45^ established a diagnostic model for the detection of hepatocellular cancer and cirrhosis.^46^ Within colorectal surgery, the microbiome has been mechanistically linked to the development of AL via the production of matrix metalloproteinase 9 by *Enterococcus* which subsequently degrades collagen resulting in tissue breakdown.^23^ Of note, in our study *Enterococcus* was only found in 6 of the samples and at relative abundances of < 0.05%. Microbiota assessed at the time of surgery in patients undergoing colorectal surgery has shown to be discriminatory for the potential development of AL as well, with patients having lower diversity and higher abundance of mucin-degrading families *Lachnospiraceae* and *Bacteroidaceae* at higher risk for AL.^20^ Longer-term, patients who suffer from post-operative complications after colorectal cancer surgery may demonstrate reduced alpha diversity up to 24 months following surgery.^47^ Recently, Hajjar et al. used a mouse model receiving FMT from colon cancer patients with and without AL after colonic resection to link-specific gut microbiota to AL and increased mucosal inflammatory cytokines.^48^ Our work here is in line with the mounting evidence demonstrating a potentially significant role of the gut microbiota in the development of colorectal surgical complications, highlighting its potential power in detecting complications prior to the development of severe clinical sequalae.

Specific bacteria are notable due to their relationship with AL. We found the genera *Alistipes* and *Clostridium sensu stricto* to be potentially protective taxa against AL, both of which are known to produce SCFA.^49,50^ SCFA, of which the most abundant are acetate, propionate, and butyrate, are microbial metabolites that participate in colonocyte health, maintenance of epithelial barrier integrity, and immunomodulation, among other functions.^51–53^ Butyrate has received particular attention in its suggested role in anastomotic healing exerting a proliferative effect on colonic epithelium.^54^ Rectal administration of butyrate in rat models of AL has been associated with improved anastomotic healing by promoting tissue repair via higher synthesis and maturation of collagen.^54,55^ We have previously shown that butyrate levels decrease significantly shortly following surgery concomitantly with a major reduction of SCFA-producing commensal genera.^56^ In particular, *Clostridium sensu stricto* has been shown to produce butyrate.^57,58^ Future studies will focus on these microbiota and associated metabolomics with measurements of SCFA. Conversely, *Akkermansia* and *Bacteroides* were associated with AL. *Akkermansia* is a potent mucin-degrading bacterium known to affect intestinal epithelial barrier function and permeability.^59–62^ *Bacteroides* may promote pathogenicity when there is a breach in the intestinal barrier, as in colon surgery, and is frequently isolated from intraabdominal abscesses.^63^ *Bacteroides* spp. can metabolize simple sugars from host cell surface glycoproteins and glycolipids at sites of infection, and possess proteases that can attack the host extracellular matrix, degrade mucin, and cleave E-cadherin of intestinal epithelium tight junctions.^63^ Importantly, the inclusion of commensal, putative protective genera in our model increased its accuracy, suggesting AL may reflect community-level processes including a breakdown in competitive exclusion that is permissive of tissue degradation.

Our study has important limitations. First, this is a preclinical murine model, which has limitations in translational potential, and requires validation in prospective human studies.^64,65^ There are a wide variety of environmental and genetic factors that impact leak formation.^66^ Furthermore, a microbial signature may be highly individualized in the clinical setting in which each person has a unique composition impacted by a multitude of factors. In this study, we present a generalized pattern identified in a highly controlled setting. It is important for future studies to address these issues by applying this index in multiple environments to determine its external validity. To enhance the identification of a pattern associated with complications, it is likely that baseline microbial composition may be included as part of a multiomics assessment, perhaps including metabolomics, metagenomics, and transcriptomics, to create a comprehensive and personalized preoperative risk assessment tool. To minimize weight loss following surgery, all mice were also exposed to DietGel prior to and following surgery. While DietGel may influence the composition of the microbiota, we have successfully utilized it in a murine model of the sleeve gastrectomy investigating role of the post-surgical microbiota in regulating the metabolic benefits of surgery.^67,68^ We elected not to pre-treat mice receiving FMT with antibiotics as this would potentially confound our results without enhancing engraftment.^69^ Despite a lack of pre-treatment, we did see low, but appreciable levels of engraftment in both WD-ldFMT and LD-wdFMT groups. We typically observe murine engraftment in the first week at levels of 40–80% when using an antibiotic conditioning protocol with either healthy or dysbiotic human donors, with greater engraftment among healthy donors.^70^ In the present study, we observed greater engraftment with the dysbiosis-associated fecal slurry; however, the almost negligible engraftment of the LD slurry was sufficient to significantly lower the frequency of AL. Patients typically receive some form of bowel preparation with or without antibiotics. Given that bowel preparation changes the microbiota and that microbiota likely plays a role in leak formation,^56,71^ it would be important to examine how these various preparations change leak rate and if the index would be informative in those situations.

In conclusion, our study found that AL creates a dysbiotic microbial community, characterized primarily by increased levels of *Bacteroides* and *Akkermansia*. We identified several key taxa that were associated with leak formation, and developed an index based on the ratio of bacteria associated with the absence to the presence of AL. This tool could have clinical implications in enabling the prediction or early detection of AL and the potential to intervene early to reduce patient morbidity and mortality. Further murine and human studies are needed to confirm these findings in a broader range of environmental and clinical settings. Our results also highlight a modifiable connection between diet, microbiota, and anastomotic healing, potentially paving the way for modulation by microbiota-targeted therapeutics at the time of surgery to reduce AL.

## Methods

### Mice and dietary intervention

C57BL/6J male mice (*n* = 64) purchased from Jackson Laboratories at 2 weeks of age were conventionally housed (Figure 1a). Mice were randomized to a 10-week feeding period of either LD (*n* = 32) or WD (*n* = 32) (see Supplemental Table S1 for detailed nutritional information). Water and chow were available *ad lib*. until the perioperative period. In developing this model, perioperative soft diet (DietGel, ClearH2O, 77-08-5022, Supplemental Table S1) was found to reduce the incidence of colonic obstruction that occurred if mice were continuously fed pelleted food and maintained body weight.^72^ Thus, all mice were transitioned to DietGel 5 days prior to surgery and were maintained on it until necropsy. Compliance with established guidelines for humane use and care of laboratory animals was carried out as approved by the University of Minnesota Institutional Animal Care and Use Committee.

### Fecal microbiota transplantation

Fecal pellets were collected and pooled from mice after 8–10 weeks of being fed LD or WD. Fecal slurries were diluted 1:5 in sterile PBS with a final concentration of 10% glycerol. Final cell counts were 1.3 × 10^9^ cells/mL (WD fecal slurry) and 3.9 × 10^7^ cells/mL (LD fecal slurry). Fecal preparations were aliquoted and stored at −80°C until the morning of surgery. Preparations were transported on ice to the operating room where mice promptly underwent gavage with 100 μL of thawed fecal slurry (LD mice received WD fecal slurry [*n* = 16], WD received LD fecal slurry [*n* = 16]). The remaining mice received 100 μL PBS gavage (LD = 16, WD = 16) to control for any effects of gavage. No antibiotics were given at any timepoints.

### Surgery, postoperative monitoring, and anastomotic scoring

Mice were fasted for up to 16 h, then placed under general anesthesia, shaved, and prepped in sterile fashion. A 2-cm low-midline incision was made to enter the abdomen. The colon was identified and transected at a point 2–3 cm from the anus. An end-to-end anastomosis was performed with interrupted 8–0 prolene sutures and anastomotic leak test was performed via saline enema to ensure that all anastomoses were sealed. Fascia and skin were each closed with running 6–0 prolene. Mice were advanced to DietGel 4 h after surgery and were maintained on this until euthanasia by CO_2_ on POD7, or sooner if they displayed clinical deterioration. Animals were checked twice daily through POD3 and daily thereafter. Food and water intake, weight, and overall health were assessed. If mice required more supervision due to clinical symptoms suggestive of AL, including reluctance to move, unkempt coat, discharge from eyes/nose, lack of balance, stumbling, stiff gait, and abdominal swelling, monitoring was increased as needed. Necropsies were performed upon euthanasia and anastomotic healing was evaluated. All anastomoses were dissected out completely at the time of necropsy to thoroughly distinguish between local adhesions and abscesses. The anastomosis was classified as 1) intact with or without adjacent adhesions, 2) contained anastomotic leak with perianastomotic abscess, or 3) gross abdominal contamination with frank dehiscence (Figure 1b). Mice that did not survive until necropsy were excluded from analysis of AL.

### DNA extraction and sequencing

Microbiota was characterized from fecal pellets collected at four timepoints: 1) following the 10-week dietary intervention, 2) DOS, 3) POD4, and 4) POD7. While the mucosa-associated microbiota may be different at the site of the anastomosis relative to expelled pellets,^73^ this can only occur at a single time point, at the time of necropsy, thus limiting clinical applicability due to the need to endoscopically insufflate the colon and obtain biopsies which may stress and disrupt the newly created anastomosis. Expelled stool remains a feasible and utilized method to assess microbial composition relative to clinical outcomes.^44^ Pellets were stored at −80°C prior to DNA extraction. DNA was extracted from individual fecal pellets (approximately 0.1 g) for microbiome analyses using the DNeasy PowerSoil Pro kit. Amplification of the V4 hypervariable region of the 16S rRNA gene was done by the University of Minnesota Genomics center using the 515F/806 R primer set followed by paired-end sequencing on the Illumina MiSeq platform (300bp).^74,75^ Amplicon sequencing depth was 35,595 reads. Sterile water controls were included on each plate. Sequence data were deposited in the NCBI SRA under accession number SRP408098.

### 16S rRNA amplicon processing and analysis

Mothur software (v.1.41.1) was used for the processing of sequence data and its analysis.^76^ Sequences were cut to 170 nucleotides (nt), paired-end joined with fastq-join and cut for quality with an average quality score of 35 over a 50 nt window, homopolymers ≤ 6 nt, no ambiguous bases, and ≤ 2 nt differences from primer sequences.^77^ Alignment of high-quality sequences was performed using SILVA database (v.138.1).^78^ Any chimeras identified were removed using UCHIME v.4.2.409.^79^ Binning of operational taxonomic units (OTUs) was done at 99% similarity using the furthest-neighbor algorithm and Ribosomal Database Project (RDP v.18) was used for taxonomic assignments.^80^ With regard to statistical comparisons, rarefication of samples was done to 35,500 reads per sample, which yielded a mean Good’s coverage estimate of 98.7 ± 0.8% among all samples. To evaluate engraftment following reciprocal FMT, we used the Bayesian algorithm SourceTracker,^81^ which determines the percent of a sink community (recipient mouse stool) that was attributable to the donor material (FMT slurry). Default parameters were used and the script was implemented in R.

### Statistical analysis

Clinical data (survival, AL, and body weight) were evaluated by ANOVA with Duncan’s *post-hoc* test for multiple comparisons, Chi-square test, or Fisher’s exact test for categorical variables where appropriate, using XLSTAT (version 2015.01.0; Addinsoft). Shannon and Chao1 indices (calculated in mothur) were used to determine alpha diversity of microbial communities and were compared using ANOVA. Kruskal-Wallis was performed to assess differences in abundances of genera and LEfSe was done to determine differences in abundance at the OTU level between experimental groups with LDA threshold > 4. Bray-Curtis dissimilarity matrices were used to calculate beta diversity and visualized by ordination using PCoA.^82,83^ These matrices were also used to assess differences in beta diversity by ANOSIM with Bonferroni correction.^84^ Relative abundances of genera were correlated to axis position using Spearman correlations, and taxa significantly correlated to group clustering were overlaid on the PCoA plot using the corr.axes command in mothur. Canonical correspondence analysis was done to visualize associations between dietary macronutrients, AL, and bacterial genera, and Spearman correlations were performed to determine statistical significance of associations. All statistics were evaluated at α = 0.05.

## Supplementary Material

Supplemental Material

## Data Availability

Sequence data were deposited in the NCBI SRA under accession number SRP408098 and can be found at https://www.ncbi.nlm.nih.gov/sra/?term=SRP408098.
