# Supplementary material for: Diet-induced shifts in the gut microbiota influence anastomotic healing in a murine model of colonic surgery
Source: Gut Microbes. 2023 Nov 22;15(2):2283147. doi: 10.1080/19490976.2023.2283147 (PMC10730186; doi:10.1080/19490976.2023.2283147)

# Supplemental Methods *FMT preparation*

Fecal pellets from each dietary group were collected from all mice after 8-10 weeks of being fed either LD (n=32) or WD (n=32), pooled, and stored at -80°C. Multiple microcentrifuge tubes containing 200 mg of stool diluted in 0.5 mL of sterile PBS were prepared, vortexed continuously for 10 minutes, then centrifuged at 500 g for 5 minutes to separate out the inorganic material. Supernatants from the same dietary groups were combined into 15 mL conical tubes and an equal volume of 20% glycerol in PBS was added to bring samples to a final concentration of 10% glycerol prior to storage at -80°C.

# Supplemental Data

**Supplemental Table 1.** Nutrition details.

| **Macronutrients** | **Lean Diet**  **(TD.08485)** | **Western Diet**  **(TD.88137)** | **DietGel**  **(72-08-5022)** |
| --- | --- | --- | --- |
| Total Kcal/g | 3.6 | 4.5 | 2.1 |
| Protein (Kcal/g) | 0.69 (19.1%) | 0.68 (15.2%) | 0.24 (11.4%) |
| Fat (Kcal/g) | 0.47 (13%) | 1.89 (42%) | 0.69 (32.9%) |
| Carbohydrate (Kcal/g) | 2.44 (67.9%) | 1.92 (42.7%) | 1.21 (57.6%) |
| Mono/Disaccharide (g/kg) | 120.2 | 341.7 | * |
| Polysaccharide (g/kg) | 583 | 200 | * |
| Fiber (g/kg) | 50 | 50 | 32 |
| Saturated fat (g/kg) | 25.4 | 132.3 | 7 |
| Monounsaturated fat (g/kg) | 13.7 | 60.9 | * |
| Polyunsaturated fat (g/kg) | 9.3 | 8.4 | * |

*Not provided in manufacturer datasheet.

**Supplemental Figure 1.** When WD FMT mice without AL were compared to WD PBS mice with AL on POD4, a slight increase in the separation of leak vs. no leak was observed and this difference was significant (ANOSIM R = 0.28, *P* = 0.03). *Not further classified to genus.


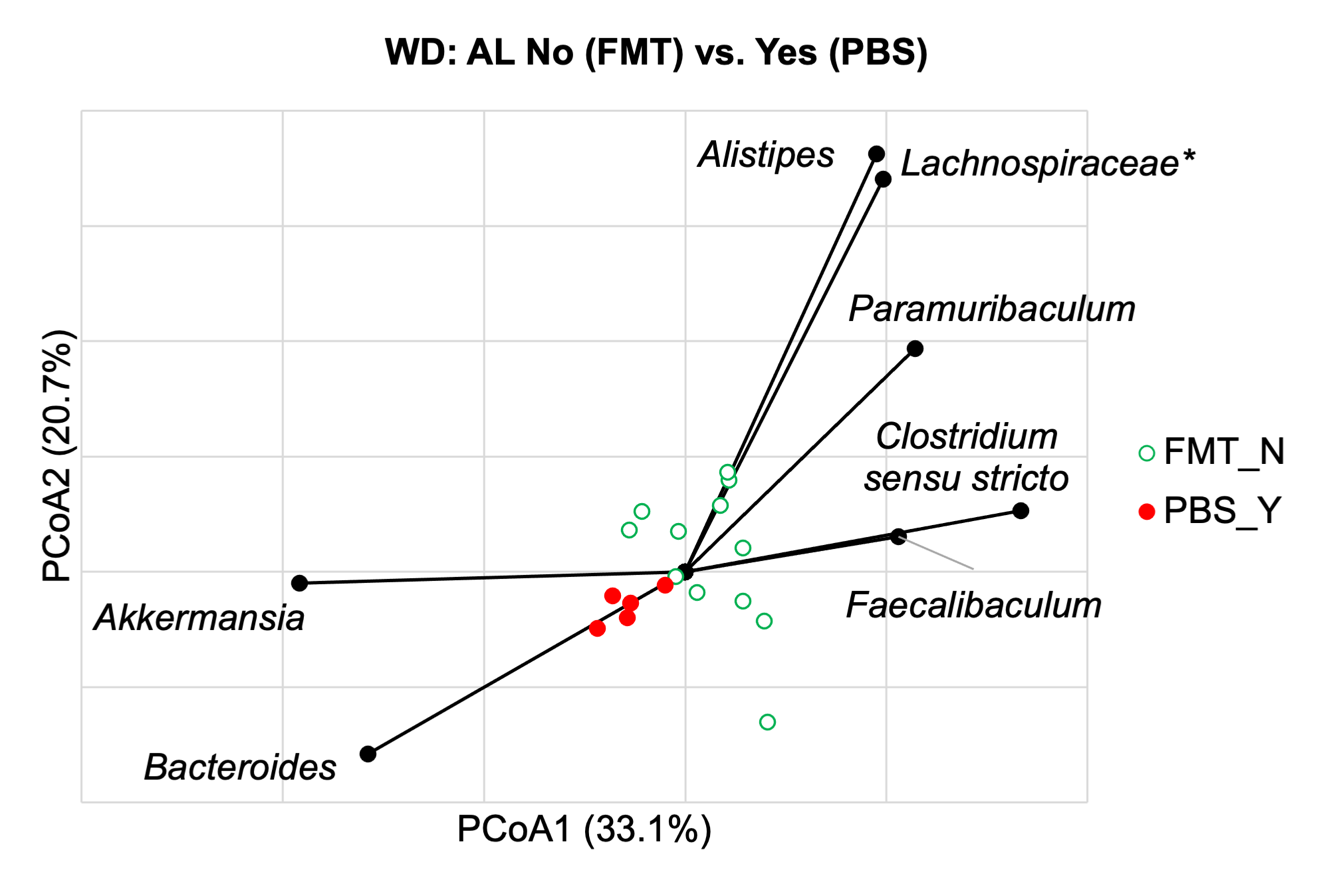


**Supplemental Figure 2.** Mean (± standard deviation) of engraftment as determined by SourceTracker2 analyses.


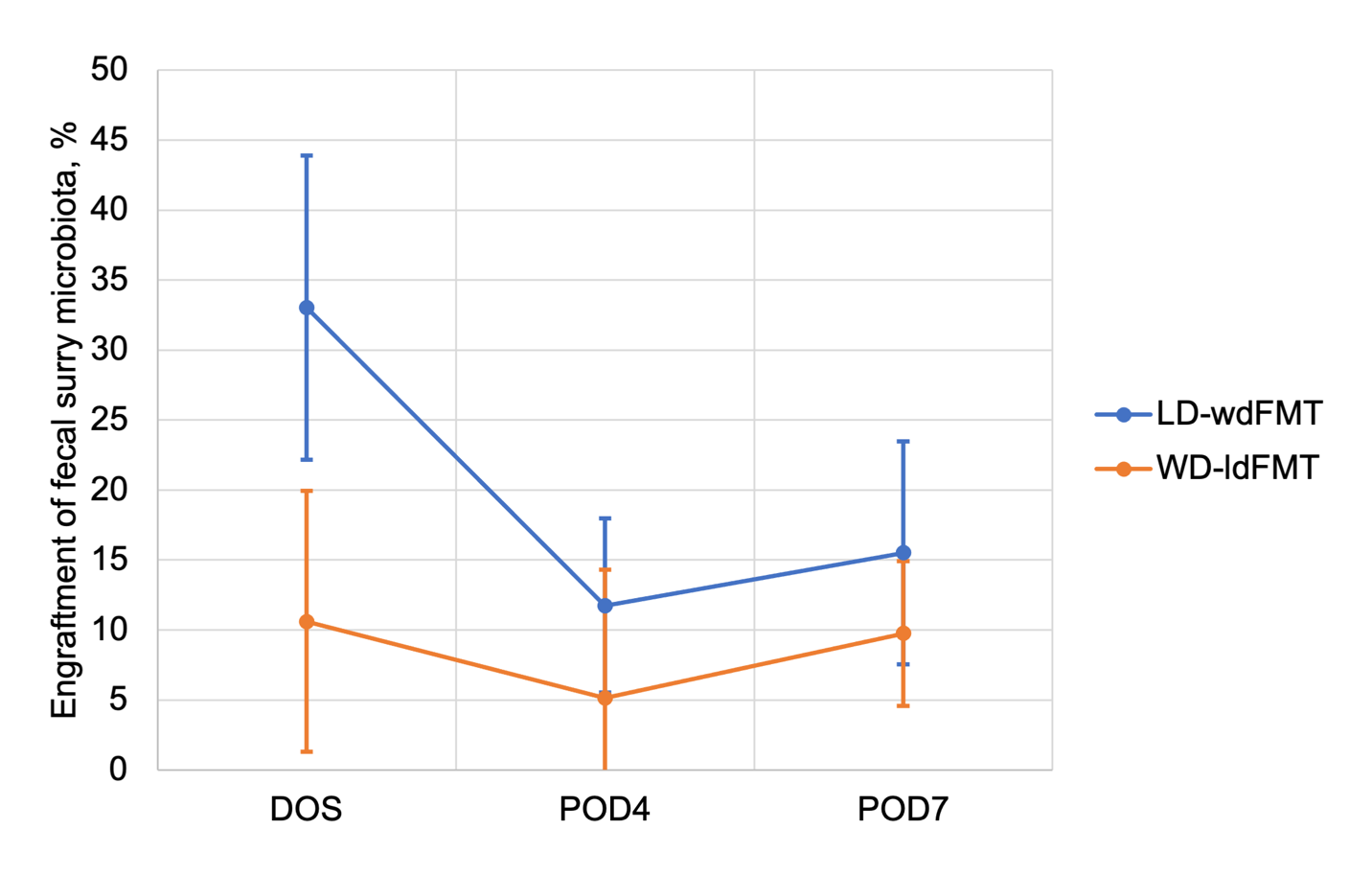


**Supplemental Figure 3.** LEfSe analysis for identification of differently abundant taxa grouped based on diet and presence of AL on POD4. The length of the vertical bar represents the LDA score in log scale, and only taxa meeting an LDA score threshold > 4.0 are listed.


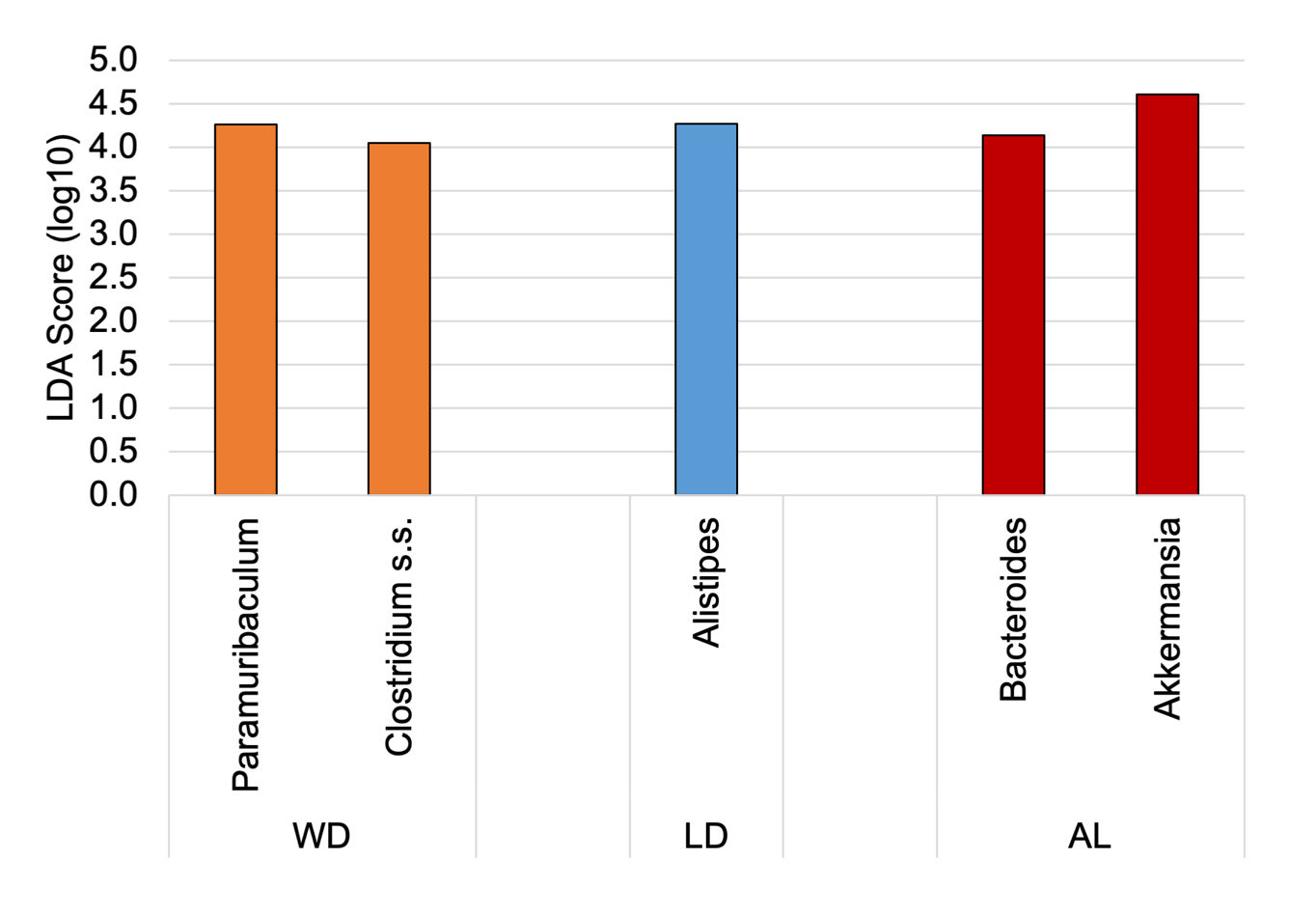

Supplement: Supplemental Material [file KGMI_A_2283147_SM0719.docx]
